# Bis(4-ferrocenylbenzoato-κ*O*)tetra­kis(methanol-κ*O*)cadmium(II)

**DOI:** 10.1107/S160053680803907X

**Published:** 2008-11-26

**Authors:** Hong-Ying Zhao, Lian-Qing Song, Jing-Wu Wang, Seik Weng Ng

**Affiliations:** a Department of Chemistry, Zhengzhou University, Zhengzhou 450052, People’s Republic of China; b Department of Chemistry, Henan Institute of Chemistry, Zhengzhou 450014, People’s Republic of China; c Department of Chemistry, University of Malaya, 50603 Kuala Lumpur, Malaysia

## Abstract

The complete mol­ecule of the title compound, [CdFe_2_(C_5_H_5_)_2_(C_12_H_8_O_2_)_2_(CH_4_O)_4_], is generated by crystallographic twofold symmetry, with the Cd atom lying on the rotation axis. The Cd atom is coordinated by the O atoms of the four methanol mol­ecules and by the O atoms of the two carboxyl­ate anions (the latter in *cis* geometry), resulting in a distorted CdO_6_ octa­hedron. The phenyl­ene ring is almost coplanar with its adjacent cyclo­penta­dienyl ring [dihedral angle = 8.2 (2)°]. The uncoordin­ated carboxyl­ate O atom acts as acceptor to two O—H⋯O hydrogen bonds from the methanol mol­ecules, giving rise to a layered network.

## Related literature

For background literature on manganese ferrocenyl-4-benzoate, see: Hou *et al.* (2004 ▶). There are no other crystallographic studies of the metal salts of this carboxylic acid.
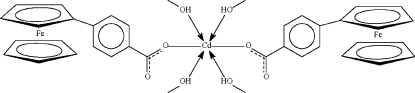


## Experimental

### 

#### Crystal data

[CdFe_2_(C_5_H_5_)_2_(C_12_H_8_O_2_)_2_(CH_4_O)_4_]
*M* *_r_* = 850.82
Orthorhombic, 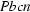

*a* = 9.8989 (4) Å
*b* = 8.6785 (4) Å
*c* = 41.228 (2) Å
*V* = 3541.8 (4) Å^3^
*Z* = 4
Mo *K*α radiation
μ = 1.46 mm^−1^
*T* = 295 (2) K
0.20 × 0.20 × 0.18 mm

#### Data collection

Bruker APEXII CCD diffractometer
Absorption correction: multi-scan (*SADABS*; Sheldrick, 1996 ▶) *T*_min_ = 0.760, *T*_max_ = 0.780
20643 measured reflections
4059 independent reflections
3059 reflections with *I* > 2σ(*I*)
*R*_int_ = 0.031

#### Refinement

*R*[*F* ^2^ > 2σ(*F* ^2^)] = 0.030
*wR*(*F* ^2^) = 0.086
*S* = 1.02
4059 reflections
232 parameters
2 restraints
H atoms treated by a mixture of independent and constrained refinement
Δρ_max_ = 0.32 e Å^−3^
Δρ_min_ = −0.80 e Å^−3^

### 

Data collection: *APEX2* (Bruker, 2007 ▶); cell refinement: *SAINT* (Bruker, 2007 ▶); data reduction: *SAINT*; program(s) used to solve structure: *SHELXS97* (Sheldrick, 2008 ▶); program(s) used to refine structure: *SHELXL97* (Sheldrick, 2008 ▶); molecular graphics: *X-SEED* (Barbour, 2001 ▶); software used to prepare material for publication: *publCIF* (Westrip, 2008 ▶).

## Supplementary Material

Crystal structure: contains datablocks global, I. DOI: 10.1107/S160053680803907X/hb2852sup1.cif

Structure factors: contains datablocks I. DOI: 10.1107/S160053680803907X/hb2852Isup2.hkl

Additional supplementary materials:  crystallographic information; 3D view; checkCIF report

## Figures and Tables

**Table:** 

Cd1—O1	2.236 (2)
Cd1—O3	2.404 (2)
Cd1—O4	2.333 (2)

**Table:** 

O1—Cd1—O1^i^	107.7 (1)

Symmetry code: (i) 
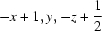
.

**Table 2:** Hydrogen-bond geometry (Å, °)

*D*—H⋯*A*	*D*—H	H⋯*A*	*D*⋯*A*	*D*—H⋯*A*
O3—H3⋯O2^ii^	0.85 (1)	1.93 (1)	2.767 (3)	172 (3)
O4—H4⋯O2^iii^	0.84 (1)	1.88 (1)	2.723 (3)	173 (3)

Symmetry codes: (ii) 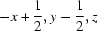; (iii) 
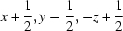
.

**Fig. 1.**
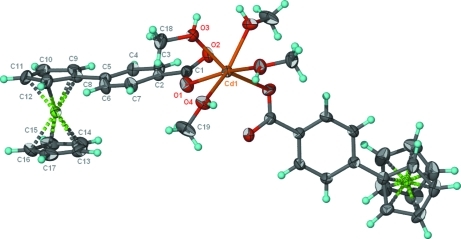
The molecular structure of (I) at the 50% probability level (arbitrary spheres for the H atoms). The unlabelled atoms are generated by the symmetry operation (1–x, 1+y, 1/2–z).

## supplementary crystallographic information

### Comment

See the Abstract for details; see Hou *et al.* (2004) for background. See Tables 1 and 2 for selected geometrical data and Fig. 1 for the molecular structure.

### Experimental

A 5-ml aqueous solution of cadmium nitrate (33.1 mg, 0.1 mmol) was mixed with an 8-ml methanol solution of sodium ferrocenyl-4-benzoate (65.9 mg, 0.2 mmol) to give a red solution. Red crystals of (I) separated afer two weeks in 60% yield.

### Refinement

The carbon-bound H atoms were generated geometrically (C–H = 0.93–98 Å), and refined as riding with *U*(H) = 1.2–1.5*U*_eq_(C). The hydroxy and water H atoms were located in a difference Fourier map, and were refined with a distance restraint of O–H = 0.85±0.01 Å; their U_iso_ values were freely refined.

### Crystal data

**Table:**

[CdFe_2_(C_5_H_5_)_2_(C_12_H_8_O_2_)_2_(CH_4_O)_4_]	*F*_000_ = 1736
*M**_r_* = 850.82	*D*_x_ = 1.596 Mg m^−^^3^
Orthorhombic, *P**b**c**n*	Mo *K*α radiation λ = 0.71073 Å
Hall symbol: -P 2n 2ab	Cell parameters from 6256 reflections
*a* = 9.8989 (4) Å	θ = 2.8–28.1º
*b* = 8.6785 (4) Å	µ = 1.46 mm^−^^1^
*c* = 41.228 (2) Å	*T* = 295 (2) K
*V* = 3541.8 (4) Å^3^	Block, red
*Z* = 4	0.20 × 0.20 × 0.18 mm

### Data collection

**Table:**

Bruker APEXII CCD diffractometer	4059 independent reflections
Radiation source: fine-focus sealed tube	3059 reflections with *I* > 2σ(*I*)
Monochromator: graphite	*R*_int_ = 0.031
*T* = 295(2) K	θ_max_ = 27.5º
φ and ω scans	θ_min_ = 2.9º
Absorption correction: Multi-scan(SADABS; Sheldrick, 1996)	*h* = −7→12
*T*_min_ = 0.760, *T*_max_ = 0.780	*k* = −11→11
20643 measured reflections	*l* = −53→49

### Refinement

**Table:**

Refinement on *F*^2^	Secondary atom site location: difference Fourier map
Least-squares matrix: full	Hydrogen site location: inferred from neighbouring sites
*R*[*F*^2^ > 2σ(*F*^2^)] = 0.030	H atoms treated by a mixture of independent and constrained refinement
*wR*(*F*^2^) = 0.086	*w* = 1/[σ^2^(*F*_o_^2^) + (0.0443*P*)^2^ + 2.0761*P*] where *P* = (*F*_o_^2^ + 2*F*_c_^2^)/3
*S* = 1.02	(Δ/σ)_max_ = 0.001
4059 reflections	Δρ_max_ = 0.32 e Å^−^^3^
232 parameters	Δρ_min_ = −0.80 e Å^−^^3^
2 restraints	Extinction correction: none
Primary atom site location: structure-invariant direct methods	

### Fractional atomic coordinates and isotropic or equivalent isotropic displacement parameters (Å^2^)

**Table:**

	*x*	*y*	*z*	*U*_iso_*/*U*_eq_	
Cd1	0.5000	0.58264 (3)	0.2500	0.03147 (9)	
Fe1	0.48719 (3)	1.31148 (4)	0.057877 (9)	0.03353 (11)	
O1	0.52537 (19)	0.7347 (2)	0.20664 (5)	0.0468 (5)	
O2	0.31729 (19)	0.8085 (2)	0.21772 (4)	0.0456 (5)	
O3	0.4522 (2)	0.3861 (3)	0.21055 (5)	0.0483 (5)	
H3	0.3698 (14)	0.361 (4)	0.2109 (8)	0.069 (11)*	
O4	0.71881 (19)	0.5056 (2)	0.23666 (5)	0.0456 (5)	
H4	0.754 (4)	0.442 (3)	0.2497 (6)	0.062 (11)*	
C1	0.4207 (3)	0.8095 (3)	0.20015 (6)	0.0336 (5)	
C2	0.4190 (2)	0.8995 (3)	0.16902 (6)	0.0305 (5)	
C3	0.3233 (3)	1.0138 (3)	0.16375 (6)	0.0359 (6)	
H3A	0.2635	1.0396	0.1803	0.043*	
C4	0.3155 (3)	1.0899 (3)	0.13431 (6)	0.0363 (6)	
H4A	0.2507	1.1663	0.1314	0.044*	
C5	0.4032 (2)	1.0539 (3)	0.10902 (6)	0.0327 (5)	
C6	0.5014 (3)	0.9418 (3)	0.11488 (7)	0.0451 (7)	
H6	0.5626	0.9172	0.0986	0.054*	
C7	0.5097 (3)	0.8672 (3)	0.14417 (7)	0.0423 (6)	
H7	0.5770	0.7940	0.1474	0.051*	
C8	0.3871 (3)	1.1246 (3)	0.07670 (6)	0.0359 (6)	
C9	0.2941 (3)	1.2435 (3)	0.06796 (7)	0.0419 (6)	
H9	0.2315	1.2957	0.0828	0.050*	
C10	0.3083 (3)	1.2742 (4)	0.03429 (7)	0.0509 (8)	
H10	0.2574	1.3510	0.0219	0.061*	
C11	0.4098 (3)	1.1772 (4)	0.02191 (7)	0.0510 (8)	
H11	0.4411	1.1738	−0.0006	0.061*	
C12	0.4585 (3)	1.0845 (3)	0.04783 (7)	0.0448 (7)	
H12	0.5298	1.0065	0.0462	0.054*	
C13	0.6253 (4)	1.3813 (4)	0.09122 (8)	0.0627 (9)	
H13	0.6439	1.3318	0.1121	0.075*	
C14	0.5318 (4)	1.4983 (4)	0.08564 (9)	0.0636 (9)	
H14	0.4725	1.5445	0.1020	0.076*	
C15	0.5368 (4)	1.5389 (4)	0.05288 (10)	0.0622 (9)	
H15	0.4815	1.6180	0.0423	0.075*	
C16	0.6327 (3)	1.4464 (4)	0.03779 (8)	0.0613 (9)	
H16	0.6572	1.4495	0.0148	0.074*	
C17	0.6896 (3)	1.3480 (4)	0.06140 (9)	0.0624 (9)	
H17	0.7609	1.2715	0.0578	0.075*	
C18	0.5144 (3)	0.3827 (4)	0.17959 (8)	0.0558 (8)	
H18A	0.5157	0.2787	0.1716	0.084*	
H18B	0.6053	0.4204	0.1813	0.084*	
H18C	0.4644	0.4465	0.1649	0.084*	
C19	0.8176 (3)	0.6002 (4)	0.22225 (10)	0.0663 (10)	
H19A	0.8713	0.5400	0.2076	0.100*	
H19B	0.8746	0.6430	0.2388	0.100*	
H19C	0.7743	0.6821	0.2105	0.100*	

### Atomic displacement parameters (Å^2^)

**Table:**

	*U*^11^	*U*^22^	*U*^33^	*U*^12^	*U*^13^	*U*^23^
Cd1	0.03018 (14)	0.03576 (14)	0.02848 (14)	0.000	0.00122 (10)	0.000
Fe1	0.0326 (2)	0.0380 (2)	0.0300 (2)	−0.00236 (15)	0.00162 (15)	0.00192 (14)
O1	0.0443 (11)	0.0510 (12)	0.0451 (11)	0.0092 (9)	0.0009 (9)	0.0168 (10)
O2	0.0476 (11)	0.0556 (12)	0.0336 (10)	0.0063 (9)	0.0098 (8)	0.0072 (9)
O3	0.0448 (11)	0.0608 (13)	0.0392 (11)	−0.0098 (10)	0.0008 (10)	−0.0061 (9)
O4	0.0352 (10)	0.0425 (11)	0.0591 (12)	0.0049 (9)	0.0004 (9)	0.0100 (10)
C1	0.0392 (14)	0.0304 (12)	0.0310 (13)	−0.0018 (11)	−0.0023 (11)	−0.0004 (10)
C2	0.0310 (12)	0.0295 (12)	0.0309 (12)	−0.0014 (9)	−0.0017 (10)	−0.0002 (10)
C3	0.0380 (13)	0.0361 (13)	0.0336 (13)	0.0061 (11)	0.0074 (11)	−0.0014 (11)
C4	0.0380 (13)	0.0344 (13)	0.0366 (14)	0.0080 (11)	0.0006 (11)	0.0008 (10)
C5	0.0320 (12)	0.0315 (12)	0.0345 (13)	−0.0027 (10)	−0.0005 (10)	0.0025 (10)
C6	0.0409 (15)	0.0577 (17)	0.0368 (14)	0.0136 (13)	0.0129 (12)	0.0057 (12)
C7	0.0351 (14)	0.0475 (15)	0.0443 (15)	0.0134 (12)	0.0062 (12)	0.0073 (12)
C8	0.0352 (13)	0.0380 (13)	0.0345 (13)	−0.0053 (11)	−0.0014 (11)	0.0008 (11)
C9	0.0316 (13)	0.0477 (15)	0.0466 (15)	−0.0030 (12)	−0.0026 (12)	0.0124 (13)
C10	0.0484 (17)	0.0598 (19)	0.0447 (16)	−0.0120 (15)	−0.0167 (14)	0.0170 (14)
C11	0.068 (2)	0.0551 (18)	0.0299 (14)	−0.0133 (16)	−0.0058 (14)	0.0002 (13)
C12	0.0604 (18)	0.0381 (14)	0.0359 (15)	−0.0024 (13)	0.0035 (13)	−0.0048 (12)
C13	0.062 (2)	0.078 (2)	0.0483 (18)	−0.0259 (19)	−0.0178 (16)	0.0046 (17)
C14	0.064 (2)	0.056 (2)	0.071 (2)	−0.0135 (17)	0.0107 (18)	−0.0210 (18)
C15	0.0586 (19)	0.0439 (17)	0.084 (3)	−0.0147 (15)	0.0019 (19)	0.0078 (18)
C16	0.057 (2)	0.074 (2)	0.0521 (19)	−0.0267 (18)	0.0129 (16)	0.0020 (17)
C17	0.0337 (15)	0.067 (2)	0.086 (3)	−0.0044 (15)	0.0020 (16)	−0.011 (2)
C18	0.063 (2)	0.066 (2)	0.0382 (16)	0.0012 (16)	0.0023 (14)	−0.0103 (15)
C19	0.0384 (17)	0.055 (2)	0.105 (3)	0.0039 (14)	0.0250 (18)	0.0073 (19)

### Geometric parameters (Å, °)

**Table:**

Cd1—O1	2.236 (2)	C5—C8	1.476 (3)
Cd1—O1^i^	2.236 (2)	C6—C7	1.373 (4)
Cd1—O3	2.404 (2)	C6—H6	0.9300
Cd1—O4	2.333 (2)	C7—H7	0.9300
Cd1—O4^i^	2.333 (2)	C8—C12	1.427 (4)
Cd1—O3^i^	2.404 (2)	C8—C9	1.429 (4)
Fe1—C13	2.031 (3)	C9—C10	1.421 (4)
Fe1—C16	2.033 (3)	C9—H9	0.9800
Fe1—C12	2.033 (3)	C10—C11	1.406 (5)
Fe1—C14	2.033 (3)	C10—H10	0.9800
Fe1—C17	2.034 (3)	C11—C12	1.422 (4)
Fe1—C11	2.036 (3)	C11—H11	0.9800
Fe1—C9	2.043 (3)	C12—H12	0.9800
Fe1—C15	2.045 (3)	C13—C14	1.392 (5)
Fe1—C10	2.046 (3)	C13—C17	1.414 (5)
Fe1—C8	2.053 (3)	C13—H13	0.9800
O1—C1	1.252 (3)	C14—C15	1.397 (5)
O2—C1	1.254 (3)	C14—H14	0.9800
O3—C18	1.418 (4)	C15—C16	1.391 (5)
O3—H3	0.845 (10)	C15—H15	0.9800
O4—C19	1.409 (3)	C16—C17	1.412 (5)
O4—H4	0.844 (10)	C16—H16	0.9800
C1—C2	1.502 (3)	C17—H17	0.9800
C2—C3	1.389 (3)	C18—H18A	0.9600
C2—C7	1.391 (4)	C18—H18B	0.9600
C3—C4	1.384 (3)	C18—H18C	0.9600
C3—H3A	0.9300	C19—H19A	0.9600
C4—C5	1.393 (3)	C19—H19B	0.9600
C4—H4A	0.9300	C19—H19C	0.9600
C5—C6	1.396 (4)		
			
O1—Cd1—O1^i^	107.7 (1)	C4—C5—C8	121.0 (2)
O1—Cd1—O4	82.9 (1)	C6—C5—C8	121.4 (2)
O1^i^—Cd1—O4	117.5 (1)	C7—C6—C5	121.5 (2)
O1—Cd1—O4^i^	117.5 (1)	C7—C6—H6	119.3
O1^i^—Cd1—O4^i^	82.9 (1)	C5—C6—H6	119.3
O4—Cd1—O4^i^	146.7 (1)	C6—C7—C2	121.0 (2)
O1—Cd1—O3	84.3 (1)	C6—C7—H7	119.5
O1^i^—Cd1—O3	159.7 (1)	C2—C7—H7	119.5
O4—Cd1—O3	79.6 (1)	C12—C8—C9	106.6 (2)
O4^i^—Cd1—O3	76.9 (1)	C12—C8—C5	126.7 (2)
O1—Cd1—O3^i^	159.7 (1)	C9—C8—C5	126.7 (2)
O1^i^—Cd1—O3^i^	84.3 (1)	C12—C8—Fe1	68.81 (15)
O4—Cd1—O3^i^	76.9 (1)	C9—C8—Fe1	69.23 (14)
O4^i^—Cd1—O3^i^	79.6 (1)	C5—C8—Fe1	128.15 (18)
O3—Cd1—O3^i^	89.6 (1)	C10—C9—C8	108.5 (3)
C13—Fe1—C16	68.09 (14)	C10—C9—Fe1	69.76 (16)
C13—Fe1—C12	121.41 (14)	C8—C9—Fe1	69.93 (15)
C16—Fe1—C12	125.04 (14)	C10—C9—H9	125.7
C13—Fe1—C14	40.07 (15)	C8—C9—H9	125.7
C16—Fe1—C14	67.42 (14)	Fe1—C9—H9	125.7
C12—Fe1—C14	156.60 (14)	C11—C10—C9	108.3 (3)
C13—Fe1—C17	40.72 (14)	C11—C10—Fe1	69.47 (17)
C16—Fe1—C17	40.63 (14)	C9—C10—Fe1	69.58 (15)
C12—Fe1—C17	107.66 (14)	C11—C10—H10	125.9
C14—Fe1—C17	67.75 (14)	C9—C10—H10	125.9
C13—Fe1—C11	156.51 (15)	Fe1—C10—H10	125.9
C16—Fe1—C11	107.45 (13)	C10—C11—C12	107.9 (3)
C12—Fe1—C11	40.90 (12)	C10—C11—Fe1	70.22 (17)
C14—Fe1—C11	161.52 (15)	C12—C11—Fe1	69.44 (16)
C17—Fe1—C11	120.81 (14)	C10—C11—H11	126.0
C13—Fe1—C9	125.32 (13)	C12—C11—H11	126.0
C16—Fe1—C9	155.81 (14)	Fe1—C11—H11	126.0
C12—Fe1—C9	68.34 (13)	C11—C12—C8	108.7 (3)
C14—Fe1—C9	108.60 (14)	C11—C12—Fe1	69.66 (17)
C17—Fe1—C9	162.21 (13)	C8—C12—Fe1	70.30 (15)
C11—Fe1—C9	68.33 (13)	C11—C12—H12	125.6
C13—Fe1—C15	67.57 (15)	C8—C12—H12	125.6
C16—Fe1—C15	39.88 (14)	Fe1—C12—H12	125.6
C12—Fe1—C15	161.55 (14)	C14—C13—C17	107.7 (3)
C14—Fe1—C15	40.06 (15)	C14—C13—Fe1	70.04 (19)
C17—Fe1—C15	67.67 (15)	C17—C13—Fe1	69.73 (18)
C11—Fe1—C15	124.74 (15)	C14—C13—H13	126.1
C9—Fe1—C15	121.60 (14)	C17—C13—H13	126.1
C13—Fe1—C10	162.06 (15)	Fe1—C13—H13	126.1
C16—Fe1—C10	120.72 (13)	C13—C14—C15	108.7 (3)
C12—Fe1—C10	68.21 (13)	C13—C14—Fe1	69.89 (19)
C14—Fe1—C10	125.59 (15)	C15—C14—Fe1	70.4 (2)
C17—Fe1—C10	155.68 (14)	C13—C14—H14	125.7
C11—Fe1—C10	40.31 (13)	C15—C14—H14	125.7
C9—Fe1—C10	40.66 (11)	Fe1—C14—H14	125.7
C15—Fe1—C10	108.23 (14)	C16—C15—C14	108.1 (3)
C13—Fe1—C8	107.75 (12)	C16—C15—Fe1	69.60 (19)
C16—Fe1—C8	162.01 (14)	C14—C15—Fe1	69.5 (2)
C12—Fe1—C8	40.89 (11)	C16—C15—H15	125.9
C14—Fe1—C8	121.47 (13)	C14—C15—H15	125.9
C17—Fe1—C8	124.86 (13)	Fe1—C15—H15	125.9
C11—Fe1—C8	68.98 (11)	C15—C16—C17	108.2 (3)
C9—Fe1—C8	40.84 (10)	C15—C16—Fe1	70.52 (18)
C15—Fe1—C8	156.46 (13)	C17—C16—Fe1	69.73 (18)
C10—Fe1—C8	68.72 (11)	C15—C16—H16	125.9
C1—O1—Cd1	112.57 (17)	C17—C16—H16	125.9
C18—O3—Cd1	122.59 (19)	Fe1—C16—H16	125.9
C18—O3—H3	115 (2)	C16—C17—C13	107.2 (3)
Cd1—O3—H3	111 (2)	C16—C17—Fe1	69.64 (18)
C19—O4—Cd1	125.27 (17)	C13—C17—Fe1	69.55 (18)
C19—O4—H4	111 (3)	C16—C17—H17	126.4
Cd1—O4—H4	115 (3)	C13—C17—H17	126.4
O1—C1—O2	123.3 (2)	Fe1—C17—H17	126.4
O1—C1—C2	117.5 (2)	O3—C18—H18A	109.5
O2—C1—C2	119.2 (2)	O3—C18—H18B	109.5
C3—C2—C7	118.0 (2)	H18A—C18—H18B	109.5
C3—C2—C1	120.8 (2)	O3—C18—H18C	109.5
C7—C2—C1	121.2 (2)	H18A—C18—H18C	109.5
C4—C3—C2	121.1 (2)	H18B—C18—H18C	109.5
C4—C3—H3A	119.4	O4—C19—H19A	109.5
C2—C3—H3A	119.4	O4—C19—H19B	109.5
C3—C4—C5	120.9 (2)	H19A—C19—H19B	109.5
C3—C4—H4A	119.5	O4—C19—H19C	109.5
C5—C4—H4A	119.5	H19A—C19—H19C	109.5
C4—C5—C6	117.5 (2)	H19B—C19—H19C	109.5
			
O1^i^—Cd1—O1—C1	−66.94 (17)	C8—Fe1—C11—C12	−37.51 (17)
O4—Cd1—O1—C1	176.39 (19)	C10—C11—C12—C8	−0.2 (3)
O4^i^—Cd1—O1—C1	24.2 (2)	Fe1—C11—C12—C8	59.66 (19)
O3—Cd1—O1—C1	96.18 (19)	C10—C11—C12—Fe1	−59.9 (2)
O3^i^—Cd1—O1—C1	169.3 (2)	C9—C8—C12—C11	−0.1 (3)
O1—Cd1—O3—C18	33.1 (2)	C5—C8—C12—C11	178.2 (2)
O1^i^—Cd1—O3—C18	160.4 (2)	Fe1—C8—C12—C11	−59.3 (2)
O4—Cd1—O3—C18	−50.7 (2)	C9—C8—C12—Fe1	59.20 (18)
O4^i^—Cd1—O3—C18	153.1 (2)	C5—C8—C12—Fe1	−122.5 (3)
O3^i^—Cd1—O3—C18	−127.5 (2)	C13—Fe1—C12—C11	−159.4 (2)
O1—Cd1—O4—C19	31.9 (3)	C16—Fe1—C12—C11	−75.5 (2)
O1^i^—Cd1—O4—C19	−74.3 (3)	C14—Fe1—C12—C11	168.4 (3)
O4^i^—Cd1—O4—C19	163.1 (3)	C17—Fe1—C12—C11	−117.0 (2)
O3—Cd1—O4—C19	117.3 (3)	C9—Fe1—C12—C11	81.5 (2)
O3^i^—Cd1—O4—C19	−150.6 (3)	C15—Fe1—C12—C11	−44.8 (5)
Cd1—O1—C1—O2	5.6 (3)	C10—Fe1—C12—C11	37.54 (18)
Cd1—O1—C1—C2	−171.99 (16)	C8—Fe1—C12—C11	119.7 (3)
O1—C1—C2—C3	−163.9 (2)	C13—Fe1—C12—C8	80.8 (2)
O2—C1—C2—C3	18.3 (4)	C16—Fe1—C12—C8	164.74 (18)
O1—C1—C2—C7	18.4 (4)	C14—Fe1—C12—C8	48.7 (4)
O2—C1—C2—C7	−159.3 (3)	C17—Fe1—C12—C8	123.29 (18)
C7—C2—C3—C4	2.1 (4)	C11—Fe1—C12—C8	−119.7 (3)
C1—C2—C3—C4	−175.6 (2)	C9—Fe1—C12—C8	−38.29 (16)
C2—C3—C4—C5	0.2 (4)	C15—Fe1—C12—C8	−164.5 (4)
C3—C4—C5—C6	−1.9 (4)	C10—Fe1—C12—C8	−82.21 (18)
C3—C4—C5—C8	174.3 (2)	C16—Fe1—C13—C14	80.5 (2)
C4—C5—C6—C7	1.4 (4)	C12—Fe1—C13—C14	−160.8 (2)
C8—C5—C6—C7	−174.8 (3)	C17—Fe1—C13—C14	118.7 (3)
C5—C6—C7—C2	0.8 (5)	C11—Fe1—C13—C14	163.9 (3)
C3—C2—C7—C6	−2.5 (4)	C9—Fe1—C13—C14	−76.4 (2)
C1—C2—C7—C6	175.2 (3)	C15—Fe1—C13—C14	37.3 (2)
C4—C5—C8—C12	−171.7 (3)	C10—Fe1—C13—C14	−42.5 (5)
C6—C5—C8—C12	4.3 (4)	C8—Fe1—C13—C14	−118.1 (2)
C4—C5—C8—C9	6.2 (4)	C16—Fe1—C13—C17	−38.1 (2)
C6—C5—C8—C9	−177.7 (3)	C12—Fe1—C13—C17	80.5 (2)
C4—C5—C8—Fe1	97.5 (3)	C14—Fe1—C13—C17	−118.7 (3)
C6—C5—C8—Fe1	−86.4 (3)	C11—Fe1—C13—C17	45.3 (4)
C13—Fe1—C8—C12	−117.8 (2)	C9—Fe1—C13—C17	164.94 (19)
C16—Fe1—C8—C12	−44.2 (5)	C15—Fe1—C13—C17	−81.3 (2)
C14—Fe1—C8—C12	−159.5 (2)	C10—Fe1—C13—C17	−161.2 (4)
C17—Fe1—C8—C12	−76.1 (2)	C8—Fe1—C13—C17	123.2 (2)
C11—Fe1—C8—C12	37.52 (18)	C17—C13—C14—C15	−0.1 (4)
C9—Fe1—C8—C12	118.3 (2)	Fe1—C13—C14—C15	−59.9 (2)
C15—Fe1—C8—C12	167.8 (3)	C17—C13—C14—Fe1	59.8 (2)
C10—Fe1—C8—C12	80.88 (19)	C16—Fe1—C14—C13	−82.3 (2)
C13—Fe1—C8—C9	123.91 (19)	C12—Fe1—C14—C13	44.9 (4)
C16—Fe1—C8—C9	−162.5 (4)	C17—Fe1—C14—C13	−38.2 (2)
C12—Fe1—C8—C9	−118.3 (2)	C11—Fe1—C14—C13	−159.6 (4)
C14—Fe1—C8—C9	82.2 (2)	C9—Fe1—C14—C13	123.2 (2)
C17—Fe1—C8—C9	165.59 (19)	C15—Fe1—C14—C13	−119.5 (3)
C11—Fe1—C8—C9	−80.77 (19)	C10—Fe1—C14—C13	165.17 (19)
C15—Fe1—C8—C9	49.5 (4)	C8—Fe1—C14—C13	80.1 (2)
C10—Fe1—C8—C9	−37.41 (17)	C13—Fe1—C14—C15	119.5 (3)
C13—Fe1—C8—C5	2.9 (3)	C16—Fe1—C14—C15	37.2 (2)
C16—Fe1—C8—C5	76.5 (5)	C12—Fe1—C14—C15	164.4 (3)
C12—Fe1—C8—C5	120.7 (3)	C17—Fe1—C14—C15	81.3 (2)
C14—Fe1—C8—C5	−38.8 (3)	C11—Fe1—C14—C15	−40.1 (5)
C17—Fe1—C8—C5	44.6 (3)	C9—Fe1—C14—C15	−117.3 (2)
C11—Fe1—C8—C5	158.2 (3)	C10—Fe1—C14—C15	−75.3 (3)
C9—Fe1—C8—C5	−121.0 (3)	C8—Fe1—C14—C15	−160.4 (2)
C15—Fe1—C8—C5	−71.5 (4)	C13—C14—C15—C16	0.5 (4)
C10—Fe1—C8—C5	−158.4 (3)	Fe1—C14—C15—C16	−59.1 (2)
C12—C8—C9—C10	0.4 (3)	C13—C14—C15—Fe1	59.6 (2)
C5—C8—C9—C10	−177.9 (2)	C13—Fe1—C15—C16	82.2 (2)
Fe1—C8—C9—C10	59.29 (19)	C12—Fe1—C15—C16	−40.7 (6)
C12—C8—C9—Fe1	−58.93 (18)	C14—Fe1—C15—C16	119.5 (3)
C5—C8—C9—Fe1	122.8 (3)	C17—Fe1—C15—C16	38.0 (2)
C13—Fe1—C9—C10	164.7 (2)	C11—Fe1—C15—C16	−74.9 (3)
C16—Fe1—C9—C10	47.2 (4)	C9—Fe1—C15—C16	−159.1 (2)
C12—Fe1—C9—C10	−81.3 (2)	C10—Fe1—C15—C16	−116.4 (2)
C14—Fe1—C9—C10	123.4 (2)	C8—Fe1—C15—C16	165.2 (3)
C17—Fe1—C9—C10	−161.6 (4)	C13—Fe1—C15—C14	−37.3 (2)
C11—Fe1—C9—C10	−37.17 (18)	C16—Fe1—C15—C14	−119.5 (3)
C15—Fe1—C9—C10	81.2 (2)	C12—Fe1—C15—C14	−160.3 (4)
C8—Fe1—C9—C10	−119.7 (3)	C17—Fe1—C15—C14	−81.5 (2)
C13—Fe1—C9—C8	−75.6 (2)	C11—Fe1—C15—C14	165.6 (2)
C16—Fe1—C9—C8	166.9 (3)	C9—Fe1—C15—C14	81.4 (3)
C12—Fe1—C9—C8	38.33 (16)	C10—Fe1—C15—C14	124.1 (2)
C14—Fe1—C9—C8	−116.93 (19)	C8—Fe1—C15—C14	45.7 (4)
C17—Fe1—C9—C8	−41.9 (5)	C14—C15—C16—C17	−0.7 (4)
C11—Fe1—C9—C8	82.49 (18)	Fe1—C15—C16—C17	−59.8 (2)
C15—Fe1—C9—C8	−159.11 (18)	C14—C15—C16—Fe1	59.1 (2)
C10—Fe1—C9—C8	119.7 (3)	C13—Fe1—C16—C15	−80.8 (2)
C8—C9—C10—C11	−0.5 (3)	C12—Fe1—C16—C15	165.4 (2)
Fe1—C9—C10—C11	58.9 (2)	C14—Fe1—C16—C15	−37.3 (2)
C8—C9—C10—Fe1	−59.39 (18)	C17—Fe1—C16—C15	−119.0 (3)
C13—Fe1—C10—C11	−164.1 (4)	C11—Fe1—C16—C15	123.7 (2)
C16—Fe1—C10—C11	80.7 (2)	C9—Fe1—C16—C15	47.9 (4)
C12—Fe1—C10—C11	−38.08 (17)	C10—Fe1—C16—C15	81.7 (3)
C14—Fe1—C10—C11	163.60 (19)	C8—Fe1—C16—C15	−160.7 (4)
C17—Fe1—C10—C11	46.7 (4)	C13—Fe1—C16—C17	38.2 (2)
C9—Fe1—C10—C11	−119.8 (3)	C12—Fe1—C16—C17	−75.6 (2)
C15—Fe1—C10—C11	122.6 (2)	C14—Fe1—C16—C17	81.6 (2)
C8—Fe1—C10—C11	−82.19 (18)	C11—Fe1—C16—C17	−117.3 (2)
C13—Fe1—C10—C9	−44.3 (5)	C9—Fe1—C16—C17	166.9 (3)
C16—Fe1—C10—C9	−159.52 (19)	C15—Fe1—C16—C17	119.0 (3)
C12—Fe1—C10—C9	81.69 (19)	C10—Fe1—C16—C17	−159.3 (2)
C14—Fe1—C10—C9	−76.6 (2)	C8—Fe1—C16—C17	−41.7 (5)
C17—Fe1—C10—C9	166.5 (3)	C15—C16—C17—C13	0.6 (4)
C11—Fe1—C10—C9	119.8 (3)	Fe1—C16—C17—C13	−59.7 (2)
C15—Fe1—C10—C9	−117.6 (2)	C15—C16—C17—Fe1	60.3 (2)
C8—Fe1—C10—C9	37.58 (17)	C14—C13—C17—C16	−0.3 (4)
C9—C10—C11—C12	0.5 (3)	Fe1—C13—C17—C16	59.7 (2)
Fe1—C10—C11—C12	59.4 (2)	C14—C13—C17—Fe1	−60.0 (2)
C9—C10—C11—Fe1	−58.9 (2)	C13—Fe1—C17—C16	−118.4 (3)
C13—Fe1—C11—C10	167.8 (3)	C12—Fe1—C17—C16	123.7 (2)
C16—Fe1—C11—C10	−117.20 (19)	C14—Fe1—C17—C16	−80.8 (2)
C12—Fe1—C11—C10	119.0 (3)	C11—Fe1—C17—C16	80.9 (2)
C14—Fe1—C11—C10	−46.4 (5)	C9—Fe1—C17—C16	−162.3 (4)
C17—Fe1—C11—C10	−159.58 (19)	C15—Fe1—C17—C16	−37.3 (2)
C9—Fe1—C11—C10	37.49 (17)	C10—Fe1—C17—C16	47.6 (4)
C15—Fe1—C11—C10	−76.8 (2)	C8—Fe1—C17—C16	165.49 (19)
C8—Fe1—C11—C10	81.48 (18)	C16—Fe1—C17—C13	118.4 (3)
C13—Fe1—C11—C12	48.8 (4)	C12—Fe1—C17—C13	−117.9 (2)
C16—Fe1—C11—C12	123.8 (2)	C14—Fe1—C17—C13	37.6 (2)
C14—Fe1—C11—C12	−165.4 (4)	C11—Fe1—C17—C13	−160.7 (2)
C17—Fe1—C11—C12	81.4 (2)	C9—Fe1—C17—C13	−43.9 (5)
C9—Fe1—C11—C12	−81.50 (19)	C15—Fe1—C17—C13	81.1 (2)
C15—Fe1—C11—C12	164.26 (19)	C10—Fe1—C17—C13	166.0 (3)
C10—Fe1—C11—C12	−119.0 (3)	C8—Fe1—C17—C13	−76.1 (2)

Symmetry codes: (i) −*x*+1, *y*, −*z*+1/2.

### Hydrogen-bond geometry (Å, °)

**Table:**

*D*—H···*A*	*D*—H	H···*A*	*D*···*A*	*D*—H···*A*
O3—H3···O2^ii^	0.85 (1)	1.93 (1)	2.767 (3)	172 (3)
O4—H4···O2^iii^	0.84 (1)	1.88 (1)	2.723 (3)	173 (3)

Symmetry codes: (ii) −*x*+1/2, *y*−1/2, *z*; (iii) *x*+1/2, *y*−1/2, −*z*+1/2.
